# Molecular Impacts of Occupational Exposure to Waste Anesthetic Gases

**DOI:** 10.1002/em.70029

**Published:** 2025-08-25

**Authors:** Mariane A. P. Silva, Mónica Cappetta, Tony F. Grassi, Leandro G. Braz, Mariana G. Braz

**Affiliations:** ^1^ GENOTOX Laboratory São Paulo State University—UNESP, Medical School Botucatu SP Brazil; ^2^ Departamento de Genética Universidad de la República—UDELAR, Facultad de Medicina Montevideo Uruguay

**Keywords:** comet assay, DNA methylation, gene expression, inhalational anesthetic, occupational exposure

## Abstract

Millions of healthcare professionals are occupationally exposed to waste anesthetic gases (WAGs) worldwide; however, their effect on DNA methylation is unknown. Thus, for the first time, global DNA methylation analysis was performed in addition to the comet assay and gene expression analysis in this case–control study to reveal possible molecular alterations associated with WAG exposure. A total of 86 participants were included (43 healthcare professionals exposed to WAGs and 43 matched‐controls). Mononuclear cells were isolated for the comet assay, and DNA was extracted from whole blood for global DNA methylation analysis. RNA was isolated for the gene expression of *NRF2*, *SIRT1*, and *HO1*. Increased DNA damage was observed in the exposed group (*p* = 0.02). No differences in global DNA methylation or *HO1* expression were observed (*p* > 0.05), but *SIRT1* (*p* = 0.01) and *NRF2* (*p* = 0.02) upregulation were observed in the exposed group. These findings highlight the molecular effects of WAG exposure.

## 1 Introduction

Waste anesthetic gases (WAGs) are volatile agents used in anesthesia that are released into the surgical room during their administration in both human and veterinary procedures. While essential for inducing and maintaining general anesthesia, their release raises significant concerns regarding occupational exposure and environmental pollution (Silva et al. 2025).

Despite increasing evidence of the genotoxic potential of WAGs, little is known about their influence on epigenetic regulation. While genotoxicity directly reflects DNA damage, epigenetic modifications can modulate gene expression related to DNA repair and the oxidative stress response, potentially influencing the persistence and severity of genetic damage (Gibney and Nolan 2010).

DNA methylation, in particular, plays a crucial role in regulating the adaptive responses of an organism to various environmental conditions (Martin and Fry 2018). Previous studies have highlighted the impact of occupational exposure to various chemicals, including heavy metals, persistent organic pollutants, endocrine‐disrupting chemicals, polycyclic aromatic hydrocarbons, tobacco, and air pollution, on DNA methylation levels (Ruiz‐Hernandez et al. 2015). However, no study to date has focused on the effects of occupational exposure to WAGs on DNA methylation, indicating a gap in the literature. Importantly, epigenetic changes are often reversible, making them potential targets for risk mitigation.

In contrast, gene expression is highly dynamic and influenced by various factors, including the time point of observation (Jaenisch and Bird 2003). Among the key regulators of epigenetic control is sirtuin 1 (SIRT1), a deacetylase that modulates chromatin structure and gene transcription through the deacetylation of histone and nonhistone proteins, including the transcription factor nuclear factor erythroid 2‐related factor 2‐NRF2 (Singh and Ubaid 2020). By deacetylating NRF2, SIRT1 enhances its stability and transcriptional activity, thereby promoting the expression of a suite of cytoprotective genes involved in redox homeostasis. One such downstream target is heme oxygenase‐1 (HO‐1), a well‐characterized NRF2‐responsive enzyme that catalyzes the degradation of heme into free iron, collectively contributing to the attenuation of oxidative stress and inflammation (Sethi et al. 2025). Moreover, this *SIRT1–NRF2–HO‐1* axis plays a critical role in maintaining genomic stability and cellular resilience in the face of environmental insults. Notably, no study has evaluated *SIRT1* expression in relation to occupational exposure to WAGs.

Therefore, we aimed to integrate global DNA methylation analysis, comet assay, and gene expression analysis to obtain a more comprehensive understanding of the molecular alterations associated with occupational exposure to WAGs.

## 2 Materials and Methods

### 2.1 Subjects

Eighty‐six subjects were included in this case–control study after approval by the Research Ethics Committee of the institution (#30055120.7.0000.5411). The exposed group (*n* = 43) consisted of healthcare professionals working in operating theaters of two large public hospitals in the countryside of São Paulo, Brazil, who were mainly exposed to isoflurane and sevoflurane (modern halogenated anesthetics) and nitrous oxide (N_2_O). This group included anesthesiologists, surgeons, and technicians with at least 1 year of occupational exposure to WAGs. Participants with infectious or malignant diseases, pregnant women, individuals who were using illicit drugs, taking medications/supplements, recently exposed to x‐rays, or consuming large amounts of alcohol (> 9 drinks/week for women and > 14 drinks/week for men) were excluded. The control group (*n* = 43) included healthy volunteers with no history of WAGs exposure, and the same exclusion criteria used for the exposed group were applied to this group.

All the participants provided written informed consent and completed a detailed questionnaire, which was structured into thematic blocks covering characteristics (sex, race, date of birth, age, weight, height, and place of origin), occupational information (job type; if exposed to WAGs, radiation, pesticides, solvents, and so on, in the workplace and frequency/years of exposure), dietary habits (consumption and frequency of meals/day, fruits, vegetables and legumes, milk and dairy products, red meat, poultry, fish, processed meats, sweets, fried foods, soft drinks and artificial juices, etc.), and lifestyles/health status (physical activity—type and frequency; smoking status and use of hookah, alcohol, drug, and toxic substance consumption; use of vitamins, antioxidants, and medications; recent infections; malignant diseases/history of chemotherapy or radiotherapy; surgeries or anesthesia; exposure to radiation, etc.).

### 2.2 Sample Collection and Leukocyte Counts

Peripheral blood samples were collected simultaneously from individuals in both groups in a fasted state during the morning to minimize potential biases. All the samples were coded and immediately processed under yellow light. The comet assay and DNA methylation analysis were performed in duplicate, while the gene expression analysis was conducted in triplicate. Additionally, differential leukocyte counts (lymphocytes and neutrophils) were determined using an XT‐2000 hematology analyzer (Sysmex Corporation, Japan). The environmental concentrations of the WAGs were determined using an infrared spectrophotometer, following the methods of a previous study (Souza et al. 2021).

### 2.3 Global DNA Methylation Analysis

DNA methylation levels were quantified by detecting 5‐methyl 2′‐deoxycytidine (5 mdC) peaks via high‐performance liquid chromatography (HPLC), as described in a previous study (Cappetta et al. 2015). Briefly, DNA was extracted from whole blood and then hydrolyzed using nuclease P1 and alkaline phosphatase to yield 2′‐deoxymononucleosides, which were separated via HPLC and detected by UV light. A mixture of standard deoxynucleosides, including 5‐methyl‐2′‐deoxycytidine was used for calibration. Global genomic DNA methylation was calculated by comparing the 5 mdC peak area relative to the total cytidine (methylated and unmethylated) peak areas.

### 2.4 Comet Assay

Peripheral blood mononuclear cells (PBMCs) were isolated using Ficoll‐Paque density gradient centrifugation, and the alkaline comet assay was performed according to a previously described protocol (Tice et al. 1991), with slight modifications and following established guidelines (Møller et al. 2020). All the samples were prepared and scored in a blinded fashion.

A base layer of 1.5% normal agarose was first applied to the slides. A mixture containing 120 μL of 0.5% low‐melting‐point agarose and 10 μL of PBMCs was carefully pipetted onto the slide, which was covered with a coverslip and left to solidify at 4°C. The coverslips were subsequently removed, and the slides were immersed in freshly prepared cold lysis buffer at 4°C (2.5 M NaCl, 100 mM EDTA, 10 mM Tris, pH 10, supplemented with 1% Triton X‐100 and 10% DMSO) for 120 min. Then, the slides were transferred to a horizontal electrophoresis tank containing cold freshly prepared alkaline buffer (300 mM NaOH and 1 mM EDTA, pH > 13) for 20 min. Electrophoresis was conducted at 0.8 V/cm and 300 mA for 20 min at 4°C. In addition to the control and exposed group slides, positive control slides (PBMCs were treated with hydrogen peroxide from Merck at 100 μM for 30 min at 4°C) were processed simultaneously in each run to ensure that electrophoresis was conducted properly (increased DNA damage expected) and to ensure reproducibility between the runs/batches.

The slides were subsequently neutralized in 0.4 M Tris–HCl (pH 7.5), followed by absolute ethanol. Slides were stained with SYBR Gold and immediately analyzed using a fluorescence microscope (400× magnification) connected to an image analysis system (Comet Assay IV, Perceptive Instruments, UK). A total of 50 nucleoids were analyzed per slide, with a total of 100 nucleoids per participant (two slides per individual), and DNA damage was quantified by measuring the tail intensity (% DNA in tail). The median % DNA in tail was calculated for each slide, and the mean of the median values was then calculated.

### 2.5 Relative Gene Expression

RNA was isolated using a PAXgene Blood RNA Kit according to the manufacturer's protocol. The integrity was assessed via electrophoresis on a 1.5% agarose gel using Tris/borate/EDTA buffer, while the RNA concentration and purity were determined using a NanoDrop One spectrophotometer (Thermo Fisher Scientific, USA). Complementary DNA (cDNA) was synthesized from RNA using a high‐capacity cDNA reverse transcription kit (Applied Biosystems—ABI, USA), following the manufacturer's instructions. Real‐time quantitative polymerase chain reaction was performed in an ABI Prism 7500 Fast thermal cycler (ABI, USA) using the TaqMan system. The target genes analyzed were sirtuin 1 (*SIRT1*) (Hs01009006_m1), nuclear factor erythroid 2‐related factor 2 (*NRF2*) (Hs00975960_m1) and heme oxygenase‐1 (*HO1*) (Hs01110250_m1). The endogenous control gene β‐actin (*ACTB*) (Hs99999903_m1) was used for normalization. A negative control (no cDNA) was added to each plate to ensure no contamination, and for the internal control, a pool of cDNA samples from healthy subjects was used; all controls were run simultaneously with the two groups of samples throughout the experiments, as previously described (Braz et al. 2011). The relative quantification (RQ) of the obtained RNA was performed via the cycle threshold (Ct) values with the formula 2^−ΔΔCt^. For each sample, the ΔCt value was determined by subtracting the mean of the triplicate Ct values of the gene of interest from the mean of the triplicate Ct values of the reference gene (endogenous control). To determine the ΔΔCt value, the ΔCt value of each sample was subsequently subtracted from the mean ΔCt value of the internal control. This last value was added to the formula 2^−ΔΔCt^ to obtain the RQ for the three analyzed genes for both analyzed groups.

### 2.6 Statistical Analysis

The sample size was calculated, and a minimum of 42 subjects per group was necessary, considering the probability of a type I error (*α*) of 5% and a type II error (*β*) of 5% (power of 95%). To account for possible missing data or study dropouts, 43 subjects were allocated into each group. Data distribution was assessed, and since all variables followed a parametric distribution, comparisons between groups were conducted via t tests. Additionally, multivariate analysis via a multiple linear regression model was performed to evaluate DNA methylation levels. For the control group, the model included age and lymphocyte and neutrophil counts as covariates. For the exposed group, the same variables were considered, with the addition of exposure to WAGs in hours/week and years. Statistical significance was set at *p* < 0.05.

## 3 Results

Table 1 shows the characteristics of the study population and the differential leukocyte counts (*p* > 0.05). The participants consisted of healthy volunteers (control group) and healthcare professionals (exposed group) with occupational exposure to WAGs (means of isoflurane: 8 ppm; sevoflurane: 10 ppm; and N_2_O: 140 ppm).

**TABLE 1:** Demographic and clinical information of the study population and occupational exposure data.

Variable	Control (*n* = 43)	Exposed (*n* = 43)	*p*
Age (years)	35.7 ± 15.0	34.6 ± 13.5	0.73
Sex (female/male)	22/21	18/25	0.20
BMI (kg/m^2^)	25.5 ± 4.8	25.4 ± 4.3	0.88
Lymphocytes (%, range: 10–40)	30.3 ± 8.3	35.0 ± 9.3	0.11
Neutrophils (%, range: 20–70)	61.3 ± 8.9	56.3 ± 10.0	0.12
Exposure to WAGs (h/week)	—	29.0 ± 19.3	—
Exposure to WAGs (years)	—	9.7 ± 12.2	—

*Note*: The data are expressed as the means ± standard deviations or absolute numbers.

Abbreviations: BMI: body mass index; WAG: waste anesthetic gases.

A significant increase in % DNA in tail, a biomarker of genotoxicity, was detected in the exposed group compared with the control group (Table 2; *p* < 0.05). With respect to epigenetic alterations, no differences in global DNA methylation levels were observed between the groups, as shown in Table 2 (*p* > 0.05). The multivariate analysis of DNA methylation in the control group did not present a good fit model (*p* > 0.05; data not shown). Similarly, the model also showed no significant association in the exposed group (*p* > 0.05; data not shown).

**TABLE 2:** Genotoxicity and epigenetic markers between groups.

Parameter	Control (*n* = 43)	Exposed (*n* = 43)	*p*
% DNA in tail	8.9 ± 6.8; 5.8 [2.5; 13.2]	16.8 ± 15.0; 11.5 [4.4; 24.7]	**0.02**
Global DNA methylation (%)	3.0 ± 0.3	3.0 ± 0.3	0.52

*Note*: The results are presented as both the means ± standard deviations and medians [25th–75th percentiles] for the comet assay and as the means ± standard deviations for methylation. Bold *p* value represents statistically significant difference of DNA damage when the exposed group was compared to the control group.

The RQ levels (calibrated with an internal control) of *SIRT1*, *NRF2*, and *HO1* in leukocytes are shown in Table 3. Both *SIRT1* and *NRF2* were significantly upregulated in the exposed group (*p* < 0.05); whereas *HO1* expression was not significantly different between the groups.

**TABLE 3:** Comparison of relative quantification in leukocytes between groups.

Relative gene expression	Control (*n* = 43)	Exposed (*n* = 43)	*p*
*SIRT1*	1.2 ± 0.4	1.9 ± 0.8	**0.01**
*NRF2*	0.9 ± 0.3	1.5 ± 0.9	**0.02**
*HO1*	1.1 ± 0.3	1.1 ± 0.4	0.49

*Note*: The results are expressed as the means ± standard deviations. Bold *p* values represent statistically significant differences in the exposed group when compared to the control group.

## 4 Discussion

Our study revealed that professionals were exposed to WAGs that exceeded the limits established (2 ppm for halogenated and 25 ppm for N_2_O) by the U.S. National Institute for Occupational Safety and Health (NIOSH 2007). There was an association between high exposure to WAG levels and DNA damage and changes in the expression of important genes but without modulation of global DNA methylation.

Our findings regarding lymphocyte and neutrophil counts align with those of a study conducted on Iranian health professionals who were exposed to high concentrations of sevoflurane, isoflurane, and N_2_O, which revealed no significant alterations in these cell counts compared with those in the control group (Neghab et al. 2023). Similarly, a study conducted with Brazilian veterinarians exposed to waste isoflurane reported no changes in lymphocyte or neutrophil counts (Grassi et al. 2025). These findings suggest that occupational exposure to WAGs may not be associated with alterations in lymphocyte and neutrophil counts. Importantly, the lack of impact on these cells minimized possible bias in the three analyses performed, especially in the global DNA methylation analysis, as variations in white blood cell counts can influence methylation status (Zhu et al. 2012).

Our findings suggest that the comet assay is an effective biomarker of occupational exposure to WAGs. A recent scoping review conducted by Ladeira et al. (2024) reported that occupational exposure to WAGs can lead to DNA‐damaging effects. However, there are discrepancies in the literature; Braz et al. (2020) reported increased DNA damage levels in professionals occupationally exposed to WAGs, whereas Aun et al. (2018) reported no alterations in the comet assay results of first‐year medical residents. This lack of DNA damage could be attributed to the fact that early exposure to WAGs may not immediately affect genetic material or to repaired DNA, considering that the comet assay measures short‐term changes that may be repaired (Collins 2004) between exposure and tissue collection.

Epigenetic modifications, including DNA methylation changes, can occur independently or alongside genotoxic effects (Goodman et al. 2022). In the present study, we report for the first time that occupational exposure to high concentrations of WAGs in the workplace did not lead to alterations in the global DNA methylation profile of exposed professionals. Importantly, both groups shared similar lifestyle habits, with most participants regularly engaging in physical exercise and maintaining comparable fruit and vegetable consumption, factors known to influence DNA methylation (Zhu et al. 2012). The absence of significant global DNA methylation suggests that while WAG exposure was sufficient to induce genotoxic damage and modulate the expression of oxidative stress‐related genes in this study, it may not have been severe enough or long enough to elicit detectable global epigenetic modifications. Alternatively, epigenetic alterations may occur in a gene‐specific manner rather than at a global level, which would not be detected by the analytical approach used in this study. Future research should explore targeted epigenetic modifications in key regulatory genes to better understand the potential epigenetic impact of long‐term occupational exposure to WAGs.

Oxidative stress can generate reactive oxygen species that can interact with macromolecules, including DNA, potentially causing damage. Antioxidative and repair mechanisms that are regulated by gene expression have evolved in cells over time, and the efficiency of these mechanisms can significantly impact the degree of genomic instability. Our gene expression analysis revealed the upregulation of *SIRT1*, a gene that regulates apoptosis and energy metabolism through its role in catalyzing protein deacetylation, thereby enhancing the ability of cells to respond to oxidative stress (Brunet et al. 2004; Wang et al. 2020). Interestingly, this upregulation occurred without changes in DNA methylation patterns, suggesting that occupational exposure to WAGs is more closely linked to oxidative stress responses than to global alterations in CpG island methylation across the genome. These findings highlight the potential importance of other epigenetic mechanisms, such as microRNAs and site‐specific gene methylation, which should be explored in future studies to gain a more comprehensive understanding of the molecular and possible epigenetic impacts of WAG exposure.

Additionally, we observed the upregulation of *NRF2*, a key regulator of cellular defense against oxidative stress, particularly chemically induced oxidative stress. NRF2 activation modulates the transcription of genes involved in protective responses, and its upregulation in our study may reflect an adaptive response to chronic oxidative stress (Jaiswal 2004). Aun et al. (2023), in a longitudinal study, reported an upregulation of genes related to DNA repair and *NRF2* during and after medical residency, with a higher workload (41 h/week) than at baseline. In contrast, Souza et al. (2021), in a cross‐sectional study, reported no significant differences in the expression of these genes in professionals exposed to WAGs for over 10 years, despite a comparable workload of 36 h/week. Similarly, Silva et al. (2024) did not observe changes in the expression of genes related to mitochondrial antioxidant enzymes in professionals exposed to WAGs over a long period compared with controls but observed increased oxidative stress levels in professionals with more years of WAG exposure (Silva et al. 2023). These contrasting findings underscore the importance of both the timing of exposure and the study design, considering the dynamic nature of gene expression. The variation in outcomes suggests that different molecular mechanisms may be impacted by WAGs exposure over time, warranting further investigation.

In our study, we hypothesize that the upregulation of *NRF2* and *SIRT1* reflects a cellular response aimed at mitigating oxidative stress and maintaining redox homeostasis, potentially serving as a protective adaptation to chronic occupational exposure to WAGs. In contrast, *HO1* did not significantly differ between the groups. This gene plays a role in modulating biological processes and regulating cellular stress responses; it is involved in heme degradation and the regulation of inflammatory processes, and its expression is typically influenced by acute oxidative stress and inflammatory cytokine signaling (Pae and Chung 2009). In addition, Aun et al. (2023) reported no alterations in *HO1* expression during or at the end of medical residency (cohort study). Given that occupational exposure to WAGs usually occurs at low levels, it is likely that this exposure activates antioxidant defense mechanisms in a controlled manner without triggering an acute stress response sufficient to induce *HO1* expression. The upregulation of *NRF2* and *SIRT1*, in the absence of significant changes in *HO1* expression, supports this interpretation in our study.

In conclusion, our study suggests that occupational exposure to high concentrations of WAGs leads to DNA damage but does not affect global DNA methylation. Our findings highlight the associations between WAG exposure and DNA breaks, as well as the upregulation of genes related to cellular homeostasis (*SIRT1*) and antioxidant defense mechanisms (*NRF2*), but without affecting the acute stress response (*HO1*). These results suggest that changes in gene expression may reflect a transient response to oxidative stress, whereas epigenetic alterations, when they occur, tend to be more stable and longer lasting. Our study underscores the complex relationship between adaptive responses and potential molecular changes (DNA damage and gene expression), emphasizing the need for further research to explore the consequences of these biological changes, especially other epigenetic modifications, in individuals with prolonged exposure to WAGs.

## Author Contributions

M.G.B., L.G.B., and M.A.P.S. conceived and designed the study; M.A.P.S. obtained approval from the research ethics board. M.A.P.S., M.C., T.F.G., and M.G.B. conducted the experiments and analyzed the data. M.A.P.S. drafted the tables and manuscript, with significant intellectual contributions from M.G.B., M.C., and L.G.B. All authors reviewed and approved the final version of the manuscript.

## Conflicts of Interest

The authors declare no conflicts of interest.

## Data Availability

The data that support the findings of this study are available from the corresponding author upon reasonable request.
